# People follow motivation-structural rules when they react to synthetised sounds

**DOI:** 10.1038/s41598-024-68165-5

**Published:** 2024-07-26

**Authors:** Beáta Korcsok, Tamás Faragó, Bence Ferdinandy, Ádám Miklósi, Péter Korondi, Márta Gácsi

**Affiliations:** 1 HUN-REN-ELTE Comparative Ethology Research Group, Budapest, Hungary; 2 Neuroethology of Communication Lab, Department of Ethology, Eötvös Loránd University, Budapest, Hungary; 3 Department of Ethology, Eötvös Loránd University, Budapest, Hungary; 4 Department of Mechatronics, Faculty of Engineering, University of Debrecen, Debrecen, Hungary

**Keywords:** Human–Robot interaction, Encoded social information, Non-verbal communication, Approach-avoidance reaction, Social robotics, Acoustic communication, Human behaviour, Mechanical engineering, Animal behaviour

## Abstract

Emotionally expressive vocalizations can elicit approach-avoidance responses in humans and non-human animals. We investigated whether artificially generated sounds have similar effects on humans. We assessed whether subjects' reactions were linked to acoustic properties, and associated valence and intensity. We generated 343 artificial sounds with differing call lengths, fundamental frequencies and added acoustic features across 7 categories and 3 levels of biological complexity. We assessed the hypothetical behavioural response using an online questionnaire with a manikin task, in which 172 participants indicated whether they would approach or withdraw from an object emitting the sound. (1) Quieter sounds elicited approach, while loud sounds were associated with avoidance. (2) The effect of pitch was modulated by category, call length and loudness. (2a) Low-pitched sounds in complex sound categories prompted avoidance, while in other categories they elicited approach. (2b) Higher pitch in loud sounds had a distancing effect, while higher pitch in quieter sounds prompted approach. (2c) Longer sounds promoted avoidance, especially at high frequencies. (3) Sounds with higher intensity and negative valence elicited avoidance. We conclude that biologically based acoustic signals can be used to regulate the distance between social robots and humans, which can provide an advantage in interactive scenarios.

## Introduction

In social robotics the functions of emotions have been considered in multiple facets of human–robot interactions (e.g., facilitating interactions, providing feedback to the human partner, recognizing the affective state of the human, exhibiting emotionally expressive behaviours^1,2^), as well as the possibilities of incorporating emotional systems in the control mechanisms of the robot, enabling the robot to behave in a more flexible and adequate way in the complex human environment^3^. *Affective computing*—emerging in the ‘90s—has been the encompassing field for most of this research^4,5^, frequently reflecting a human-centric approach in the study and modelling of emotions. A compatible interdisciplinary framework, *ethorobotics*, focuses on the functional view of emotions from the perspective of ethology (the field of biology concerned with animal and human behaviour research), considering the skills of artificial agents related to affective phenomena as part of the required social competence of these agents that is needed of them to be able to fulfil their functions in the complex human environment^6,7^. In this endeavour, ethorobotics seeks to establish general rules based on biological principles that are observable across species, creating social skills that can be adapted to artificial agents with a high variety of embodiments and functions. We follow this conceptual framework in the present study.

Although there is a vast literature of emotion theories with numerous emotion definitions, the one that is more aligned with the ethorobotics approach is the definition by Nesse, in which emotions are defined as specialized modes of operation under the effects of natural selection, that support adaptive responses of the organism on the physiological, psychological and behavioural level to specific stimuli^3^, for example by eliciting approach towards or avoidance away from a stimulus, or by synchronizing the behaviour of group members via emotional contagion^8^.

Emotions and other affective phenomena are usually interpreted in two main conceptualizations. Models with discrete emotions usually demarcate basic emotions such as fear, sadness, happiness, anger, surprise etc., which is a commonly used system both in the research of human facial gestures^9^ and in social robotics^10^. In dimensional models however, the *core affect*, the experienced affective feeling is placed along various scales or dimensions^11,12^. The most frequently used dimensions are valence (from positive to negative emotions), arousal or intensity (from low to high intensity), and approach-avoidance tendencies (approaching a stimulus or withdrawing from it)^13^, although most dimensional models only utilise the valence and intensity dimensions (e.g., Russell’s circumplex model of affect^14^). In recent years new neuroscientific results promoted the development of unified approaches, in which systems of both discrete emotions and of core affect coexist and interact to fulfil separate functional roles^11^.

### Coding rules in acoustic communicative signals

The communicative roles of emotion expressions are recognised in humans and various non-human animal taxa, along with the evolutionary roots of human emotion expressions^15–17^. The shared evolutionary background gives rise to overall similarities in emotionally expressive behaviours connected to specific inner states^18^, which can be replicated in robots and virtual artificial agents as well (based on emotionally expressive behaviours of dogs^19–21^; and on multiple animal species^22^). In this vein, general rules can be formulated as guidelines connecting the features of emotionally expressive signals to the associated inner states.

In acoustic communication, this is the result of the evolutionarily conservative processes of sound production in terrestrial tetrapods via the shared similarities of the vocal tract anatomy and the physiological processes. The source-filter framework^23,24^ construes the connection between these features and the vocal parameters of the produced sounds, as well as the connection between the vocalizations and the inner state of the organism^25^. Through the evolutionary process of ritualization, sounds caused by involuntary exhalations in situations connected to specific inner states (e.g., running from a predator) are thought to have gained communicative functions and developed into more complex acoustic vocalizations^26^. These vocalizations however are still carrying the vestige of the physiological processes occurring during the initial scenarios from which they arose; vocalizations emitted in high arousal situations in general have higher pitch and are comprised of longer calls, which can be traced back to the tenseness of the respiratory muscles and the vocal folds^27^. Based on this principle, the acoustic cues present in emotionally expressive vocalisations have been found to give rise to simple coding rules, connecting e.g., the pitch and call duration parameters of vocalisations to their perceived valence and intensity^28–30^. The simple coding rules are present in various animal species, and can be observed even in artificially generated sounds, irrespectively of their biological complexity^31^. The existence of acoustic coding rules is also corroborated by a body of research demonstrating that humans are capable of correctly interpreting the expressed emotional states of various animal vocalisations^30,32–38^, including acoustic parameters beyond fundamental frequency and call length (e.g., spectral centre of gravity and harmonic-to-noise ratio^39^).

### Functional role of the acoustically encoded social information

The functional, biological roles of communicational signals can be interpreted based on the reactions exhibited by the receivers of the signal^25^. The perception of certain stimuli can lead to specific emotional inner states in an individual, which causes it to exhibit emotionally expressive behaviours, communicating its inner state to potential receivers of the signal^25^. The simple coding rules relating to the valence and intensity of emotionally expressive behaviours are not sufficient in explaining the array of behavioural responses exhibited by animals and humans that receive these communicative signals^29^. For example, vocalisations that are rated as high intensity and negative valence can occur with different inner states (e.g., screaming in fear or roaring during anger, as expressed with discrete emotional terms) caused by different stimuli or situations (e.g., an infant’s separation from its caretaker, or an agonistic interaction between an animal protecting its territory from an intruder, or between sports fans of opposing teams)^25^. In these examples even though the valence and intensity of the inner states are somewhat similar, the functional role of these vocalisations, and the expected effect on a receiver is different. During an agonistic interaction over territory the goal of the emitter is to drive away the intruder, and the intruder commonly reacts by withdrawing from the emitter. In case of a separation call, the goal of the emitter is to prompt its caretaker to return, and accordingly, the likely reaction of the receiver is to approach the emitter. These functional roles are reflected in the *social* dimension of communicative signals, indicating whether the adaptive reaction of the receiver would be to approach or avoid the emitter of the signal^40–42^. This evolutionary selection pressure is especially relevant in close interactions, where the appropriate reaction to this encoded social information can enable the animal to e.g., avoid injuries from fights or to ensure the safety of its offspring. The approach and avoidance reactions elicited in the receivers of the signals are affected by the acoustic features of the vocalisations according to Morton’s motivation-structural rules: vocalisations with wide frequency bands (noisy sounds) and low pitch are generally emitted in hostile interactions (with the function of eliciting an avoidance reaction), while narrow frequency band sounds with high pitch usually occur in appeasing (e.g., the emitter is submissive or afraid) and friendly interactions^43^. For example, rat (*Rattus norvegicus*) ultrasonic vocalisations follow similar patterns: higher frequency (~ 50 kHz) vocalisations elicit approach from conspecifics, while lower frequency (~ 22 kHz) vocalisations function as distress sounds, and prompt avoidance^44^. These higher frequency, approach-inducing sounds are also characteristically shorter than the lower frequency aversive sounds^45^.

### Social dimension in HRI

The capability of exhibiting emotionally expressive behaviours is a desired trait in social robots, and its lack can contribute to negative attitudes towards them^46^. While many robotics research focuses on the emotion expression of social robots as a communicative feature (for reviews, see Refs.^3,47,48^) or e.g., as a method to increase trust towards a robotic agent^49^, the possible approach or avoidance promoting effects of emotion expressions on human–robot interaction are less investigated (but see: investigation of affective qualities of robot movement features in industrial settings affecting human approach-avoidance reactions^50^; the effect of voice style (female/male synthesized voice, robotic synthesized voice) on approach distance towards a robot^51^). Similarly, acoustic signals are of major interest in human–machine interactions, including a wide range of applications such as sound design for vehicles^52,53^, or movement sonification for robotic agents in HRI and the effects of consequential sounds unintentionally created by moving parts^54^, e.g., various servo motors^55^. For overarching reviews of sounds that can occur or be actively produced by robots during human–robot interactions, see: Refs. ^56,57^. The social dimension could be an important aspect of acoustic communication in social robotics, as sounds that elicit approach or avoidance (withdrawal) from the emitter of the sound can have various practical implications in HRI. Acoustic signals that elicit the approach of the robot could be utilised for example in leading scenarios in which the human should follow the robot to a location^58^, when the robot encounters an unsolvable problem or experiences an error and is in need of human assistance, in situations where the robot should call the attention of the human to an object or situation (e.g., reminder to take medication), or to prompt interaction with the human. Communicational signals resulting in avoidance of the robot (or objects nearby it) could be employed in security guard roles or as warnings for areas to avoid (e.g., slippery floor).

As presented above, shared acoustic features of animal and human vocalisations can prompt functionally relevant approach-avoidance reactions in receiving individuals. However, it has not been established, how can functionally similar vocalisations be adapted to the communicational system of artificial agents. Our goal was to investigate whether corresponding approach-avoidance reactions could be elicited by artificially generated sounds. We have generated artificial sounds, starting from simple, machine-like sine-wave sounds, then added increasing levels of acoustic parameters characteristic of animal vocalisations in each category to account for varying levels of biological complexity, covering parameters such as harmonics, parameter variability, pitch contour changes and formants. The fundamental frequency and call length was systematically adjusted throughout the samples. The sound samples have also been used in a previous study, examining valence and intensity ratings of the sounds by human listeners (for further details see: Ref.^31^), in which we found that participants rated the valence and intensity of the sounds following the acoustic coding rules apparent in terrestrial tetrapod vocalisations, based on the fundamental frequency and call length of the sounds. In the current study, we intended to shed light on the social dimension aspect of artificial sounds, by measuring approach-avoidance reactions in a contextually neutral setup.

### Questions and hypotheses

Our main aim was to test whether humans can attribute encoded social information to artificially generated sounds. Such assumed social information result in functionally relevant behavioural changes, in approach or avoidance, providing a third dimension alongside the valence and intensity axes for emotionally expressive vocalisations. In this study we address two specific questions:

(1) Do specific acoustic features influence the direction of the behavioural response (approach-avoidance)?

We assume that the elicited approach-avoidance reactions are linked with acoustic features, and that due to the acoustic complexity of mammalian vocalisations, approach-avoidance reactions will be affected by the complex interactions of specific acoustic features of the generated sounds. We expect that sounds with short call length, usually associated with positive inner states, elicit approach, while sounds with high fundamental frequency linked with high arousal will elicit avoidance^31,37^. Besides, acoustic features may affect the reactions according to the motivation–structural rules, with high frequency sounds eliciting approach and low frequency sounds prompting avoidance^43^. We also predict that higher loudness will promote avoidance reactions, similarly to vocalisations emitted as e.g., alarm calls^59^.

(2) Are approach-avoidance responses linked to the perception of valence and intensity of the artificially generated sounds?

As the perception of valence and intensity is dependent on acoustic features in both animal vocalisations^29^ and artificial sounds (see our previous study^31^), we evaluated their association with the social information assessments in a separate model to avoid multicollinearity (due to the cross-correlation of acoustic parameters and emotion scales found in ^31^). We expect that sounds characterised by high intensity and negative valence elicit avoidance. Alternatively, the approach-avoidance reactions could also be independent from the perceived valence of the sounds, as vocalisations emitted in two differing situations, for example, indicative of negative valence inner state (whines during separation and growls in agonistic interactions^60^) can have opposing message for the receiver, eliciting approach and avoidance, respectively.

## Method

### Subjects

Subjects were volunteers aged 18 or more from various nationalities. They were recruited through online advertisement and did not receive monetary benefits for their participation. 80 participants filled out the questionnaire in English (55 female, 25 male, mean age = 41.13 ± 12.17 years), and 92 in Hungarian (53 female, 39 male, mean age = 37.97 ± 10.98 years). The experimental protocol has been approved by the United Ethical Review Committee for Research in Psychology (EPKEB) Budapest, Hungary, and the study was carried out in accordance with it (ethical permission reference number: 2022-76 (2019/49)). The subjects gave their informed consent to participate.

### Stimuli

**Figure 1:**
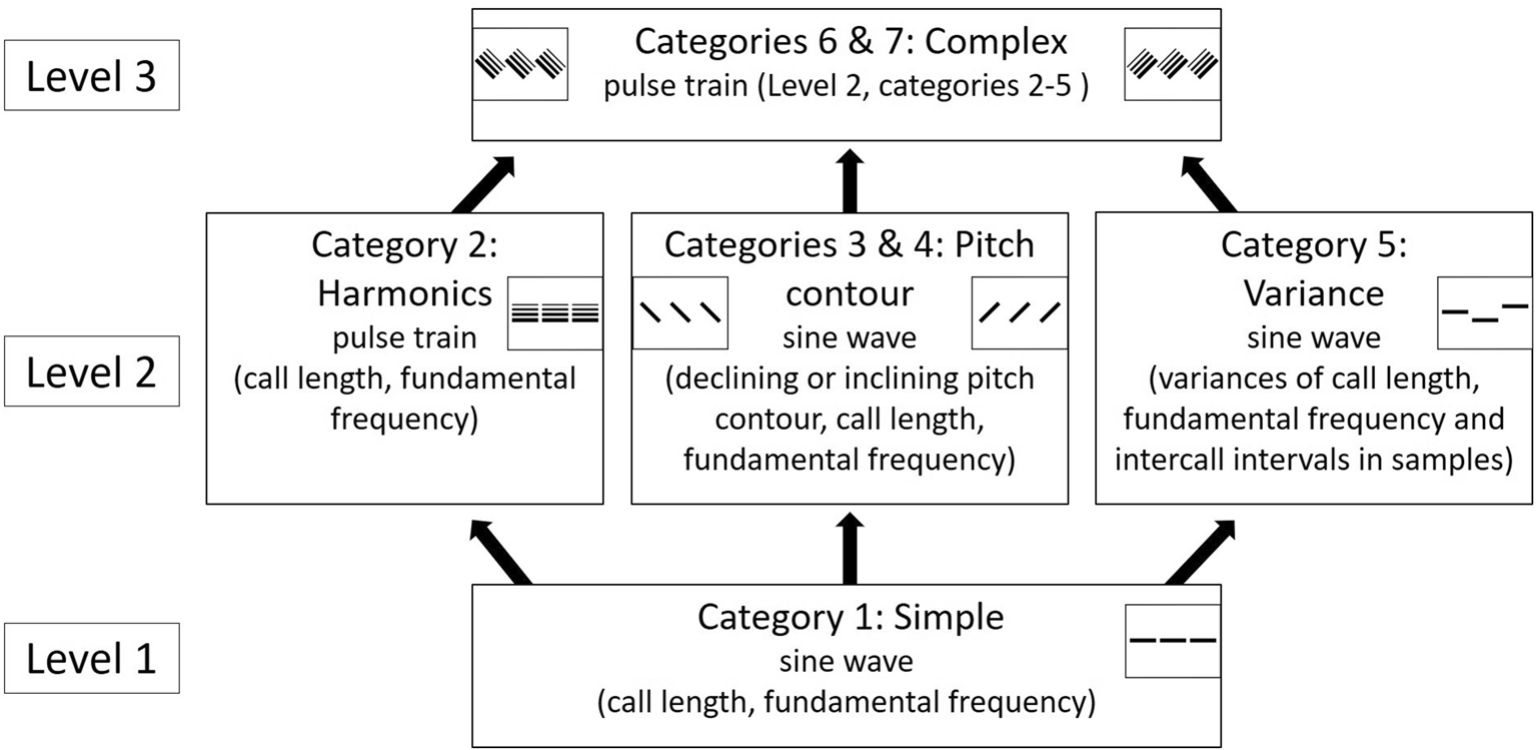
The sound categories of the artificially generated sounds. The three levels indicate the biological complexity of the sound categories. The figure has been first published in Ref.^31^.

The sound stimuli were generated in Praat (version 6.0.19) with a custom script created by TF and BK, and were the subset of the sounds used in a previous study^31^. The generated sounds consisted of multiple calls and muted inter-call periods. The call length and fundamental frequency of the calls were systematically varied. The sounds were generated in 7 complexity categories with added acoustic parameters such as harmonics, variation in acoustic parameters, pitch contour changes and formants (see Fig. 1).

49 sounds were selected from all 7 categories, resulting in 343 sounds in total. The subset of sounds was selected to cover all of the call length and frequency ranges of the generated sounds, as well as the valence and intensity ratings which were measured during our previous study^31^. The acoustic parameters and valence ratings of the sound samples are shown in Table 1, and the sound samples used in the study are available in the Supplementary Materials.

**Table 1:** Parameters of sound samples.

Parameters	Value or range (across all samples)	Variance in categories Sine_wave; Pulse_train; Pitch_down; Pitch_up	Variance in categories Variable; Complex_down; Complex_up	References
Fundamental frequency (*f*_0_)	65–1365 Hz	–	uniformly distributed random value, ± 5% of *f*_*0*_	~ 50–1600 Hz ^37^
Total length (call length + interval length)	~ 2 s (+ silence until 3 s total duration)	–	–	2 s ^37^
Call length	0.07; 0.16; 0.46; 0.76; 1.06; 1.96 s	–	uniformly distributed random value, ± 25% of call length	0.11–2 s ^37^
Valence scores	− 37.63 to 22.41	–	–	^31^
Intensity scores	14.42–77.52	–	–	^31^
Inter-call interval length	0.2 s	uniformly distributed random value, ± 25% of interval length	uniformly distributed random value, ± 50% of interval length	0.05–1.7 s ^61^
Pitch contour change in categories Pitch_down; Pitch_up; Complex_down; Complex_up	Uniformly distributed random value, ± 10% of *f*_*0*_	–	–	^43^
Vocal tract length in categories Complex_down; Complex_up	20 cm	–	–	Modelling medium sized dog ^62^
Number of formants in categories Complex_down; Complex_up	10	–	–	Modelling medium sized dog ^62^
First formant (f_1_) in categories Complex_down; Complex_up	550 Hz	–	–	Modelling medium sized dog ^62^

Table and caption adopted from ^31^.

Categories: Sine_wave: Simple sine wave; Pulse_train: Pulse train; Pitch_down: Sine wave sounds with pitch contour down; Pitch_up: Sine wave sounds with pitch contour up; Variable: Variable sine wave; Complex_down: Complex pulse train sounds with pitch contour down; Complex_up: Complex pulse train sounds with pitch contour up. More variance was implemented in the sounds of categories Variable, Complex_down and Complex_up, than in the other categories. Pitch contour changes were only present in categories Pitch_down, Pitch_up, Complex_down and Complex_up, and formants were only modelled in the categories Complex_down and Complex_up.

### Online questionnaire

The questionnaire could be accessed online at https://soundrating3.elte.hu/.

**Figure 2:**
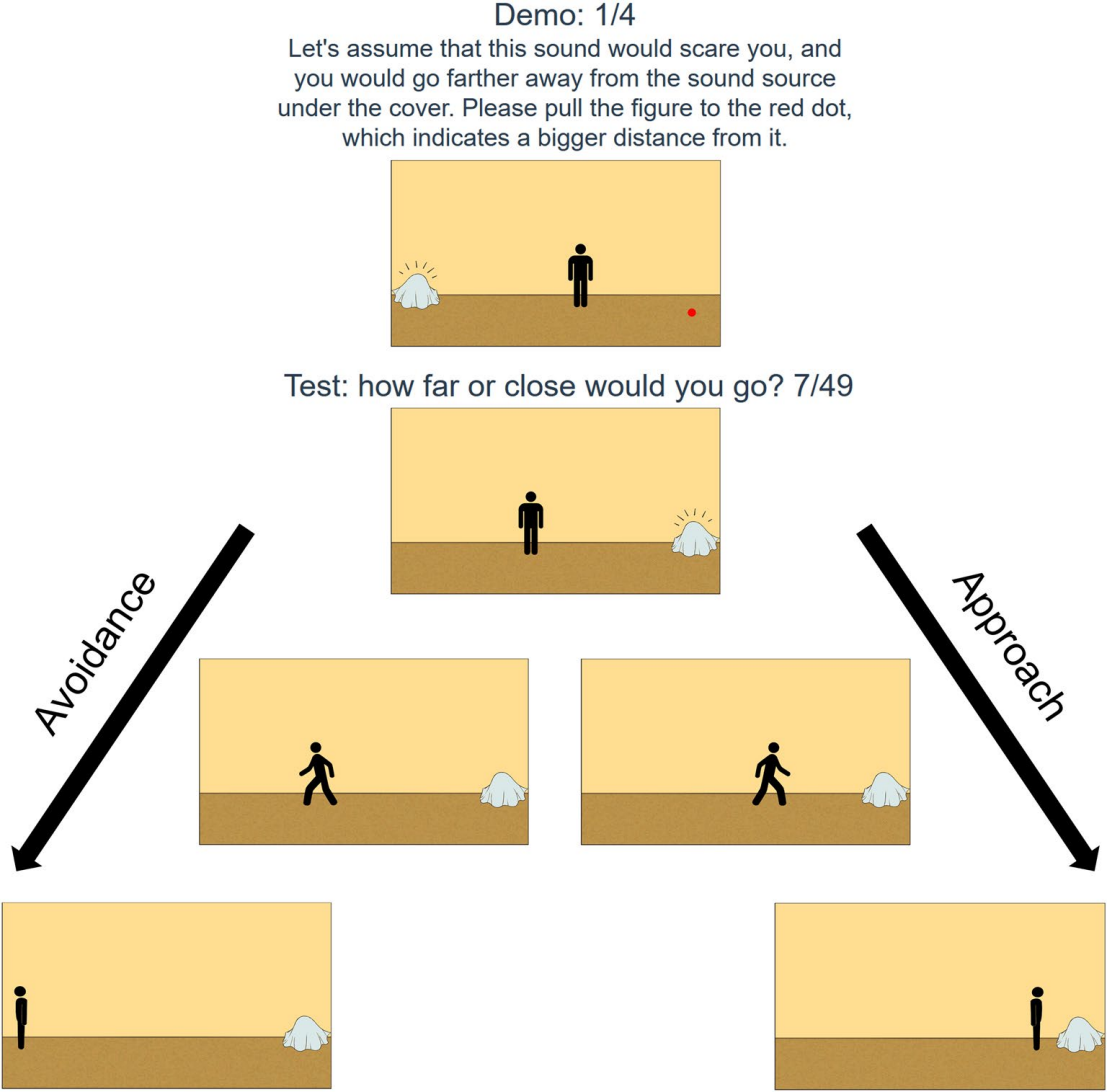
Setup of the manikin task in the online questionnaire. The figure shows a demo round and the approach-avoidance reactions enacted via the manikin. Demo round before the test: the manikin appears facing the screen while the sound is played, the lines appearing above the object indicate sound emission. Starting screens during the test: the sound emitting object appears on the left or right side of the screen. Avoidance: the manikin faces away from the object after being dragged in the opposite direction to the farthest possible point. Approach: the manikin turns to the direction of movement, then it is dragged to the object to the closest possible point.

The webpage was available in Hungarian and English. In the introductory part it offered basic information on the research and general instructions on filling out the questionnaire. These were followed by demographic questions about the participants’ gender, age and nationality, and a consent form. Next, the questionnaire started with four demo sounds explaining the participants how to indicate if they would approach or get farther away from an unknown object emitting the sound samples. The participants marked this by moving a stylized figure with their mouse or touchpad, the “manikin”, on a continuous horizontal scale closer or farther away from the object on the screen. The manikin appeared in the middle of the screen facing towards the screen, while the unknown object—which was represented as a form on the ground covered by a sheet—appeared on one side (Fig. 2). The side was randomized with the rule that the object could not appear on the same side on three consecutive occasions. After the screen loaded the sound sample was played, which was accompanied with a short animation of lines appearing above the covered form of the unknown object, signifying sound emission. The manikin, when moved, turned towards the direction of movement (Fig. 2). The participants then had to click on the next button to record the position of the manikin and move on to the next sound. Similar methods using manikins can be found in other studies measuring approach-avoidance^38^. The participants received 49 samples; 7 sounds randomly selected from each category.

### Data analysis

Statistical analysis was conducted in R statistical environment (https://www.r-project.org/) using RStudio (https://posit.co/products/open-source/rstudio/). Responses which were slower than 20 s were excluded to avoid issues caused by network problems. As the response distribution followed a very unique shape that could not be modelled by either beta, zero–one inflated beta or ordinal beta regressions, we transformed the response scale into a 5-step ordinal scale with the following levels: far (− 1 to − 0.6), medium far (− 0.6 to − 0.2), stay (− 0.2 to 0.2), medium close (0.2–0.6) and close (0.6–1). We ran mixed effects ordinal regressions (cumulative link mixed models, ordinal package, clmm function) with the derived response scale. Model fit was checked using check-model function from the easystats package. We built three separate models; one for testing demographic (age, sex), and set-up related (survey language, side of the stimulus) effects, one for acoustic effects (call length, pitch, loudness, sound category and their two-way interactions), and another model for investigating the mean valence and intensity ratings obtained from our earlier questionnaire study^31^. Each model contained participant ID and sound ID as random intercepts. In the results the models are reported with Likelihood-ratio (LR) tests. For post-hoc analysis we used emmeans and emtrends functions from emmeans package, *p* values were adjusted with Tukey method.

## Results

### Acoustic, demographic and other effects

The first model showed no significant effect of either tested demographic or setup-related effects (see Supplementary Table S1). In the second model, which tested acoustic effects, distance score was affected by sound category in interaction with pitch (LR test: χ^2^(6) = 69.837; *p* < 0.001) and loudness (LR test: χ^2^(6) = 15.059; *p* = 0.020). Pitch also affected the distance score in interaction with call length (LR test: χ^2^(1) = 13.329; *p* < 0.001) and loudness (LR test: χ^2^(1) = 28.901; *p* < 0.001) (for model details see Supplementary Table S2).

**Figure 3:**
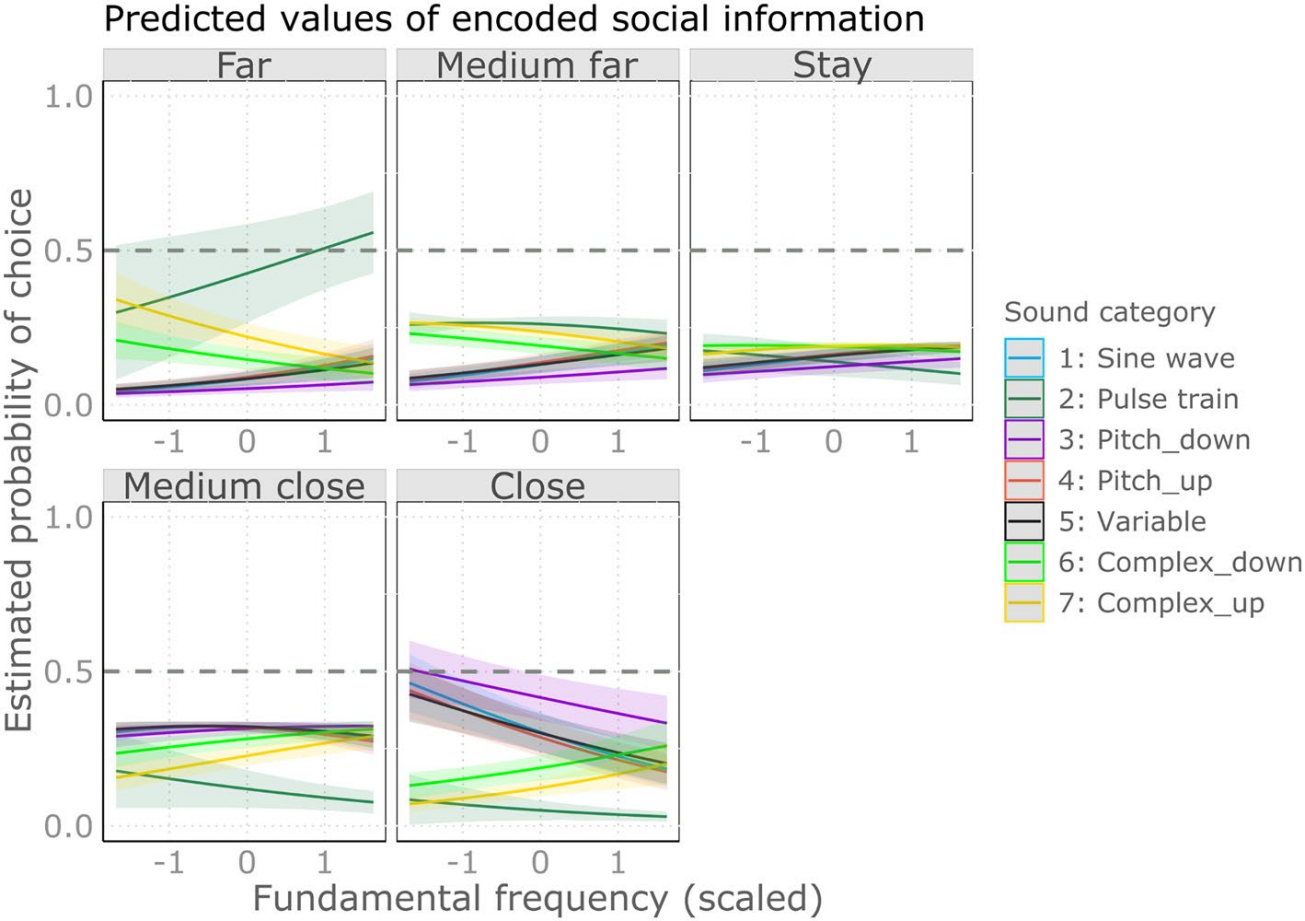
The effect of pitch on the estimated probability of choosing scale levels across the various sound categories. Categories: Sine wave: Simple sine wave; Pulse train: Pulse train; Pitch_down: Sine wave sounds with pitch contour down; Pitch_up: Sine wave sounds with pitch contour up; Variable: Variable sine wave; Complex_down: Complex pulse train sounds with pitch contour down; Complex_up: Complex pulse train sounds with pitch contour up.

Lower pitched sounds were more likely to evoke avoidance in complex sounds, while in other categories of sounds, approach. The post-hoc analysis of the pitch-sound category interaction revealed that pitch had significant positive effects on the distance scores in the complex sounds while negative in the other categories (Fig. 3). This means that in complex sounds, the lower the pitch was, the more likely the participants chose moving away from the sound, while the higher the pitch was, the more likely they chose to approach. On the contrary, in the other categories we found opposite effect. Pairwise comparisons show that negative effects (in Sine wave, Variable and Pitch categories) indeed differed significantly from the positive ones (Complex categories; see Fig. 5A) and Supplementary Table S3).

**Figure 4:**
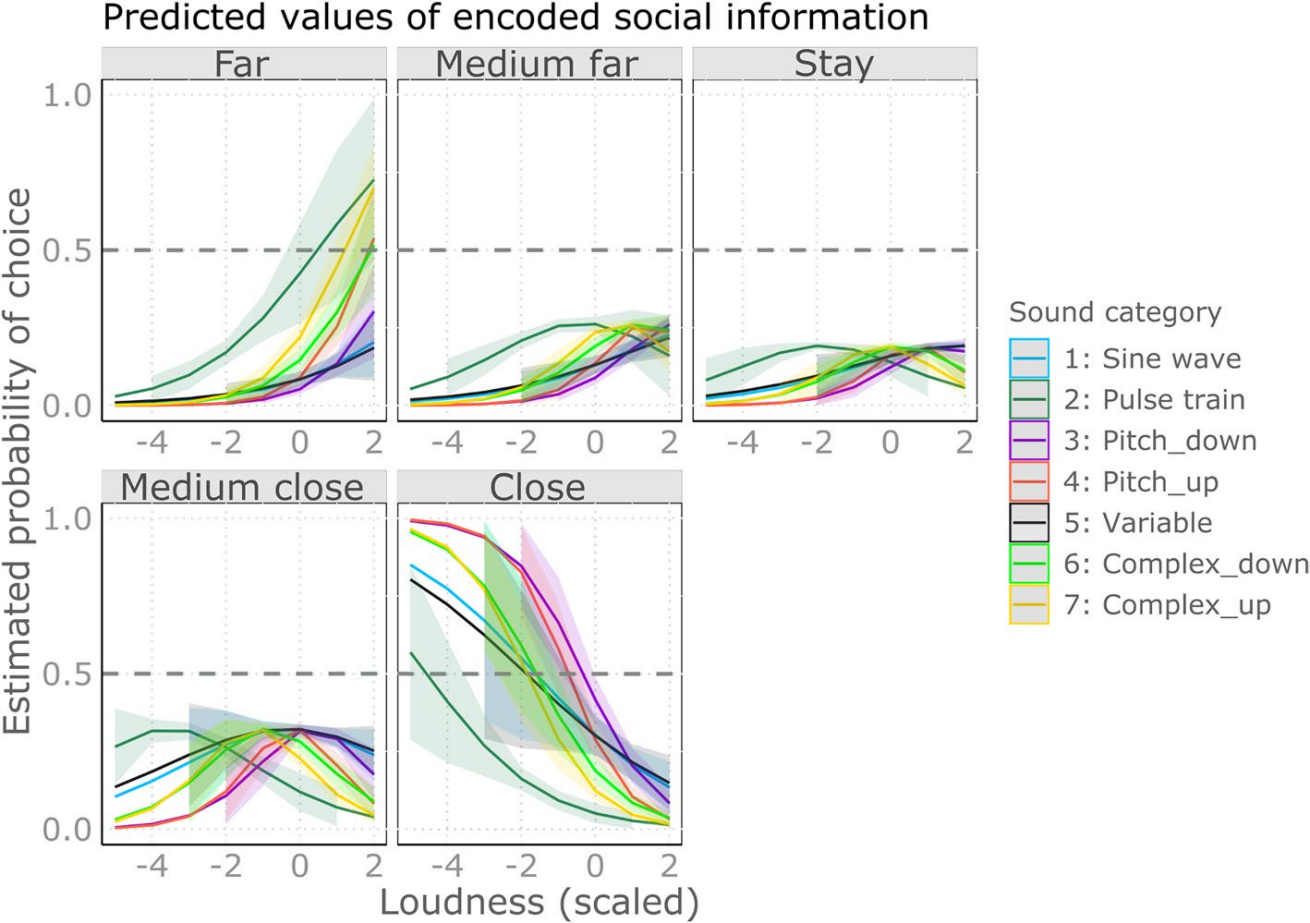
The effect of loudness on the estimated probability of choosing scale levels across the various sound categories. Categories: Sine wave: Simple sine wave; Pulse train: Pulse train; Pitch_down: Sine wave sounds with pitch contour down; Pitch_up: Sine wave sounds with pitch contour up; Variable: Variable sine wave; Complex_down: Complex pulse train sounds with pitch contour down; Complex_up: Complex pulse train sounds with pitch contour up.

**Figure 5:**
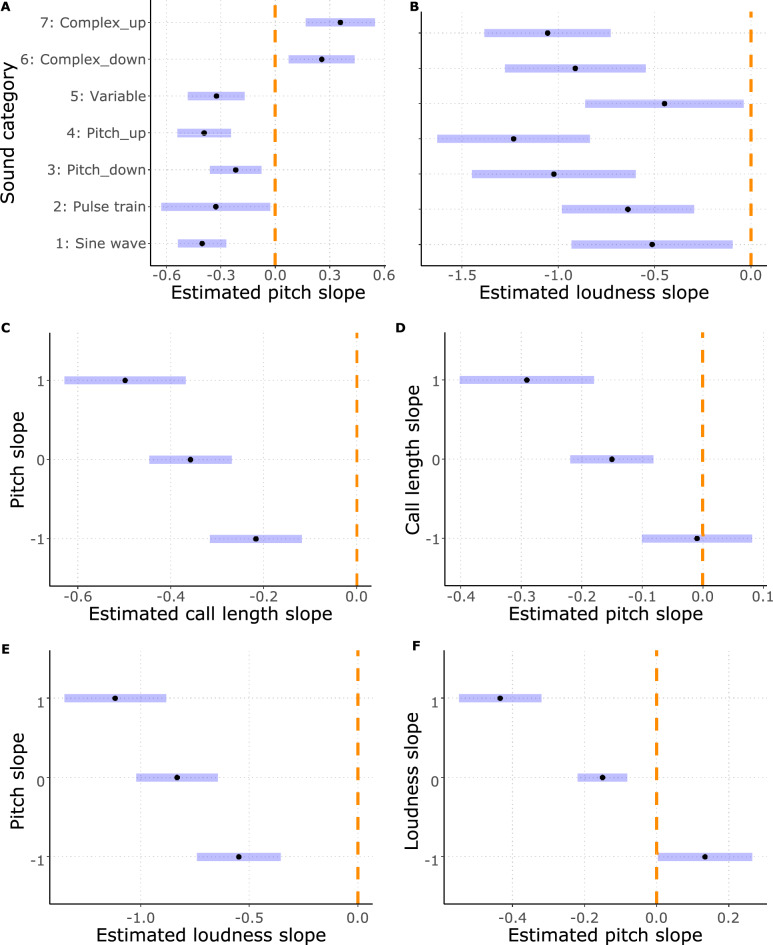
Forest plots showing estimated effect slopes (black dots) with their confidence intervals (blue stripes). The dashed orange line shows the line of no effect (zero slope, meaning a not significant effect for an estimated slope in case the confidence interval crosses the line of no effect). (**A**) Estimated pitch slope within each sound category (**B**) Estimated loudness slope within each sound category (**C**) Estimated call length slope in interaction with pitch (**D**) Estimated pitch slope in interaction with call length (**E**) Estimated loudness slope in interaction with pitch (**F**) Estimated pitch slope in interaction with loudness. Categories: Sine wave: Simple sine wave; Pulse train: Pulse train; Pitch_down: Sine wave sounds with pitch contour down; Pitch_up: Sine wave sounds with pitch contour up; Variable: Variable sine wave; Complex_down: Complex pulse train sounds with pitch contour down; Complex_up: Complex pulse train sounds with pitch contour up.

Softer sounds in general tended to evoke approach, while loud ones an avoidance: loudness had a general negative effect on the distance scores (Fig. 4). Pairwise comparisons showed no difference among the categories (see Fig. 5B) and Supplementary Table S4).

**Figure 6:**
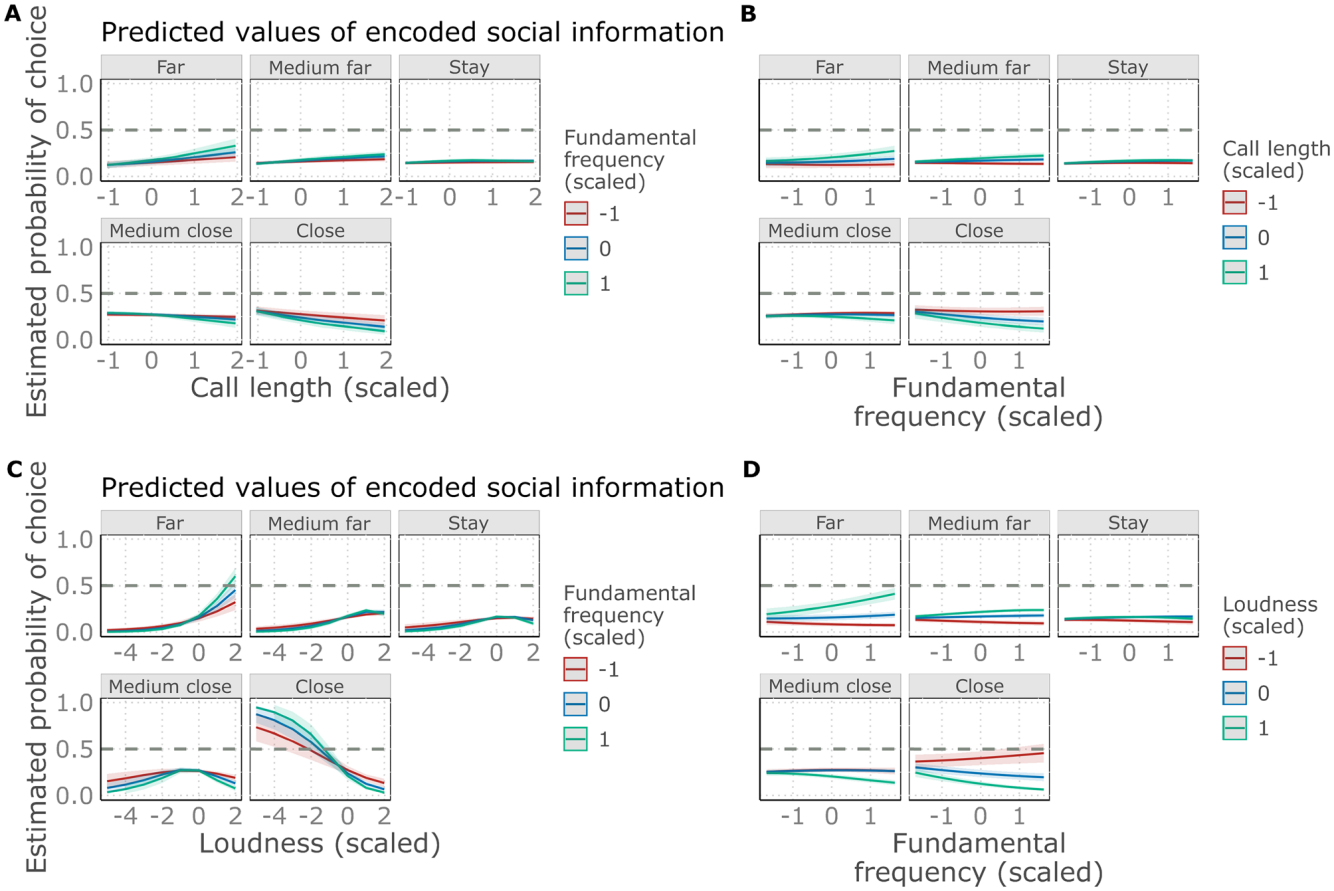
(**A**) The effect of call length on the estimated probability of choosing scale levels on the different values of pitch. (**B**) The effect of pitch on the estimated probability of choosing scale levels on the different values of call length. (**C**) The effect of loudness on the estimated probability of choosing scale levels on the different values of pitch. (**D**) The effect of pitch on the estimated probability of choosing scale levels on different values of loudness. Due to the scaling of the fundamental frequency, call length and loudness values, the mean values shown in the legends are 0, while − 1 and 1 indicates the − 1 SD and the + 1 SD respectively.

In the case of the interaction between pitch and call length we see a general negative effect of call length on the distance score, modulated by the pitch of the calls: in lower pitch sounds the call length effect is weaker, but in general longer calls tend to evoke avoidance compared to shorter ones (Figs. 5C, 6A). In contrast, in shorter calls, we see no effect of pitch on the distance score, while in longer ones, pitch has a negative effect (Figs. 5D, 6B). Irrespective of pitch, short calls evoke an approach more likely, while in long calls high pitched calls tend to evoke avoidance, and low-pitched ones evoke an approach.

In the case of the effect of pitch and loudness interaction on the distance scores we found that the negative effect of loudness is modulated by pitch: in lower sounds loudness has less strong effect (Figs. 5E, 6C). In contrast, pitch has a negative effect on the distance score in louder sounds, while this trend turns in the case of softer sounds (Figs. 5F, 6D). Low pitched sounds tend to evoke approach independently from loudness, while loud and high-pitched calls evoke avoidance, while soft and high-pitched an approach more likely.

### Emotional scale associations

**Figure 7:**
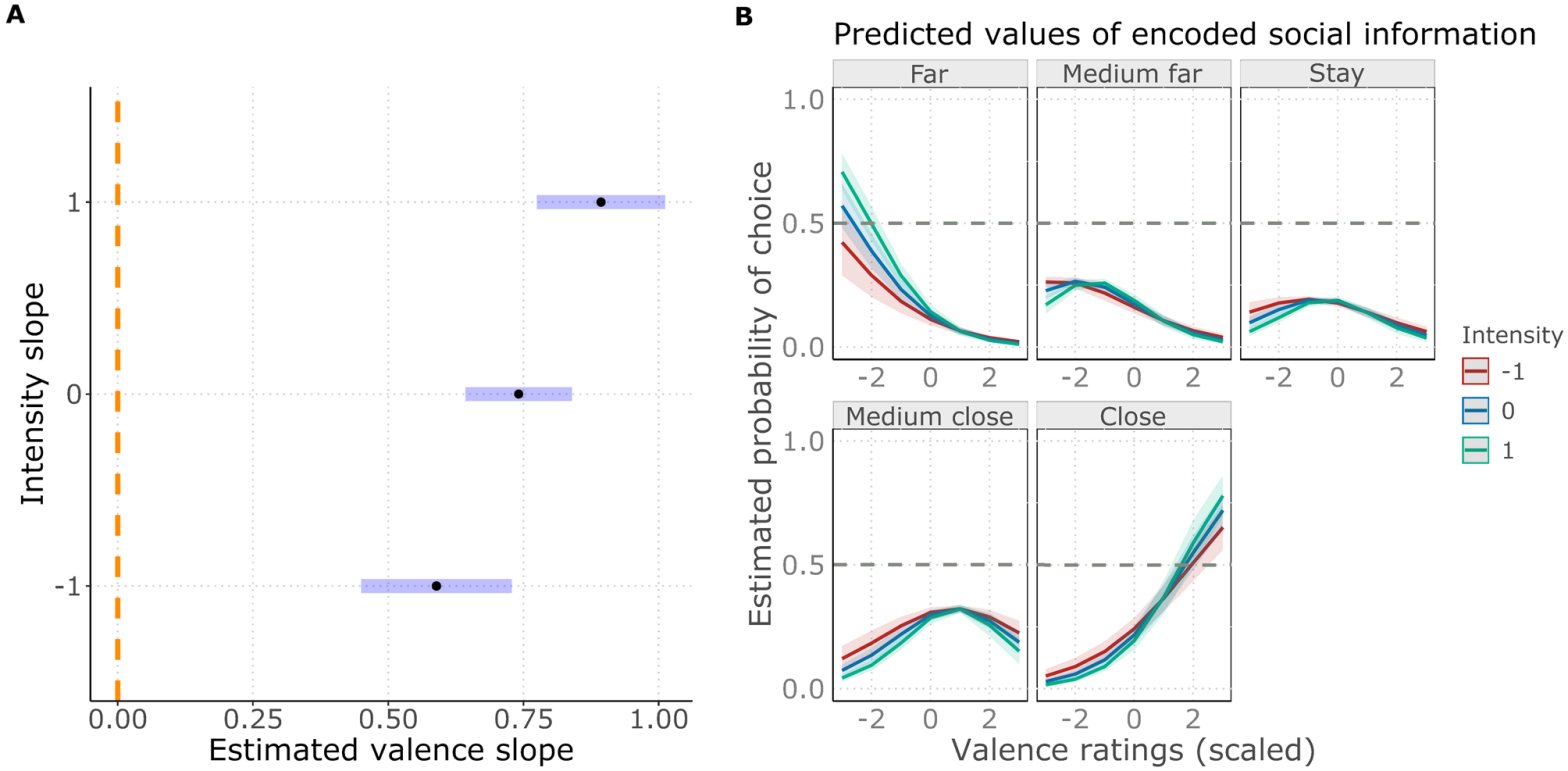
(**A**) Forest plot showing the estimated valence slopes (black dots) with their confidence intervals (blue stripes) in interaction with intensity. The dashed orange line shows the line of no effect (zero slope, meaning a not significant effect for an estimated slope in case the confidence interval crosses the line of no effect). (**B**) The effect of valence on the estimated probability of choosing scale levels, in interaction with intensity. Colours show valence slopes on the − 1 SD (red), mean (blue) and + 1 SD (green) levels of intensity.

**Figure 8:**
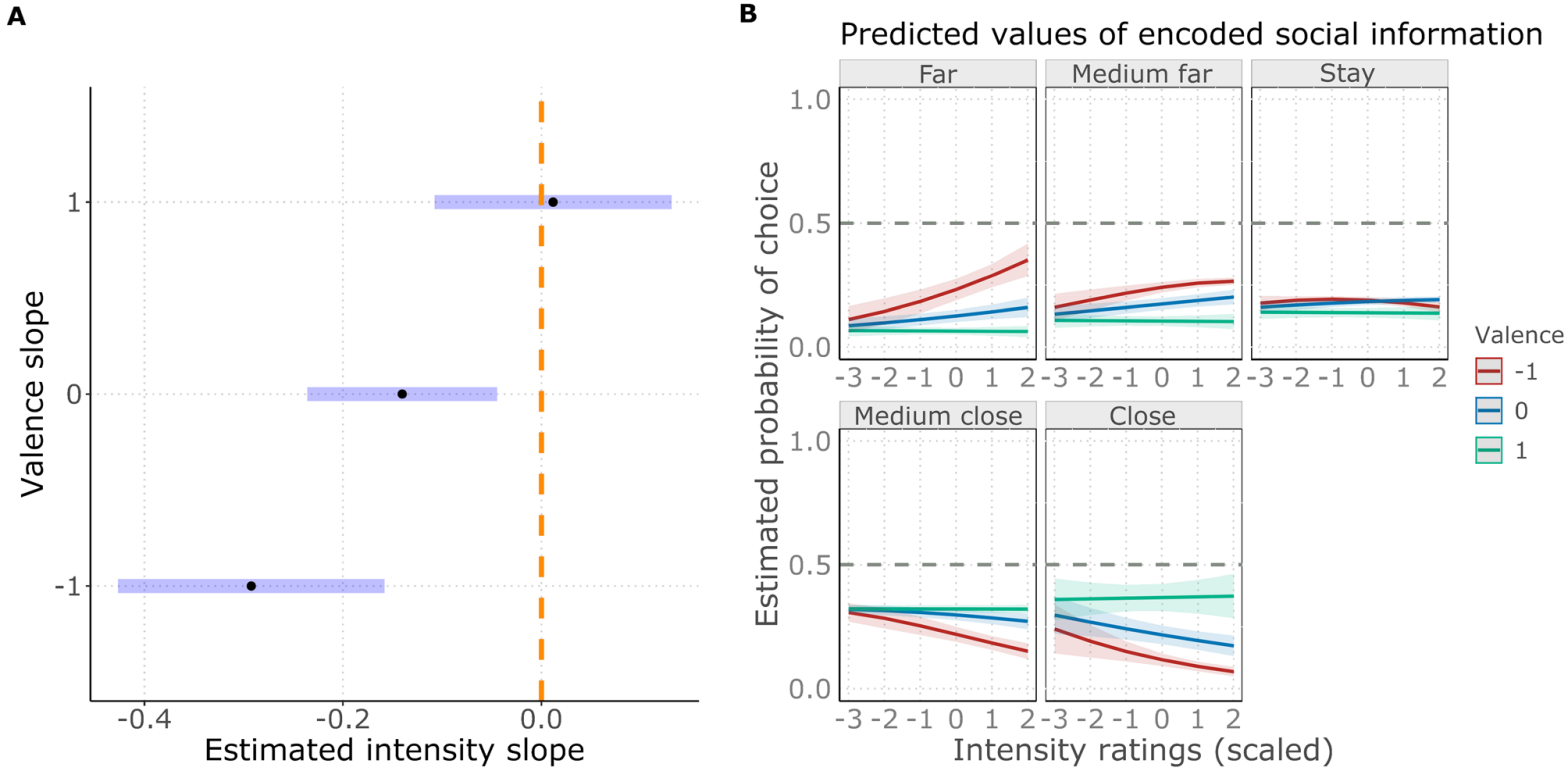
(**A**) Forest plot showing the estimated intensity slopes (black dots) with their confidence intervals (blue stripes) in interaction with valence. The dashed orange line shows the line of no effect (zero slope, meaning a not significant effect for an estimated slope in case the confidence interval crosses the line of no effect). (**B**) The effect of intensity on the estimated probability of choosing scale levels, in interaction with valence. Colours show intensity slopes on the − 1 SD (red), mean (blue) and + 1 SD (green) levels of valence.

We found that valence and intensity ratings were associated with distance scores in a significant interaction (LR test: χ^2^(1) = 12.497; *p* < 0.001; for details, see: Supplementary Table S5). Valence overall was associated positively with the distance scores: sounds rated to be more positive evoked approach, sounds rated to be negative evoked avoidance more likely, but this was somewhat positively modulated by the perceived intensity too: in more intense sounds the positive valence association was stronger (Fig. 7A,B). In contrast, intensity had a negative association with the distance scores, but this was significant only in sounds rated to be neutral or more negative (Fig. 8A,B). Sounds perceived to be aroused and negative evoke avoidance, while low-intensity sounds, independently from their valence, tend to evoke an approach. Sounds perceived to be positive, regardless of their intensity evoke approach.

## Discussion

To model the complexity of animal vocalisations we generated artificial sounds by modulating multiple acoustic features, enabling the exploration of their main and interactive effects on the social dimension. Based on our results people react to artificially generated sounds with acoustically driven behavioural responses of approach or avoidance on all complexity levels.

In the present study we investigated the effect of artificial sounds in a neutral context scenario, with an undefined agent and situation, in order to exclude possible influences of the embodiment features of a displayed robot (e.g., human-likeness, preconceptions of subjects on various robots based on their appearance). Using an undefined agent or object as a sound source is a frequently applied solution in bioacoustics research in animal studies, to investigate the effects of the acoustic features of sounds in themselves (see e.g., Ref.^63^ in which the sounds were emitted from inside a covered cage).

**Figure 9:**
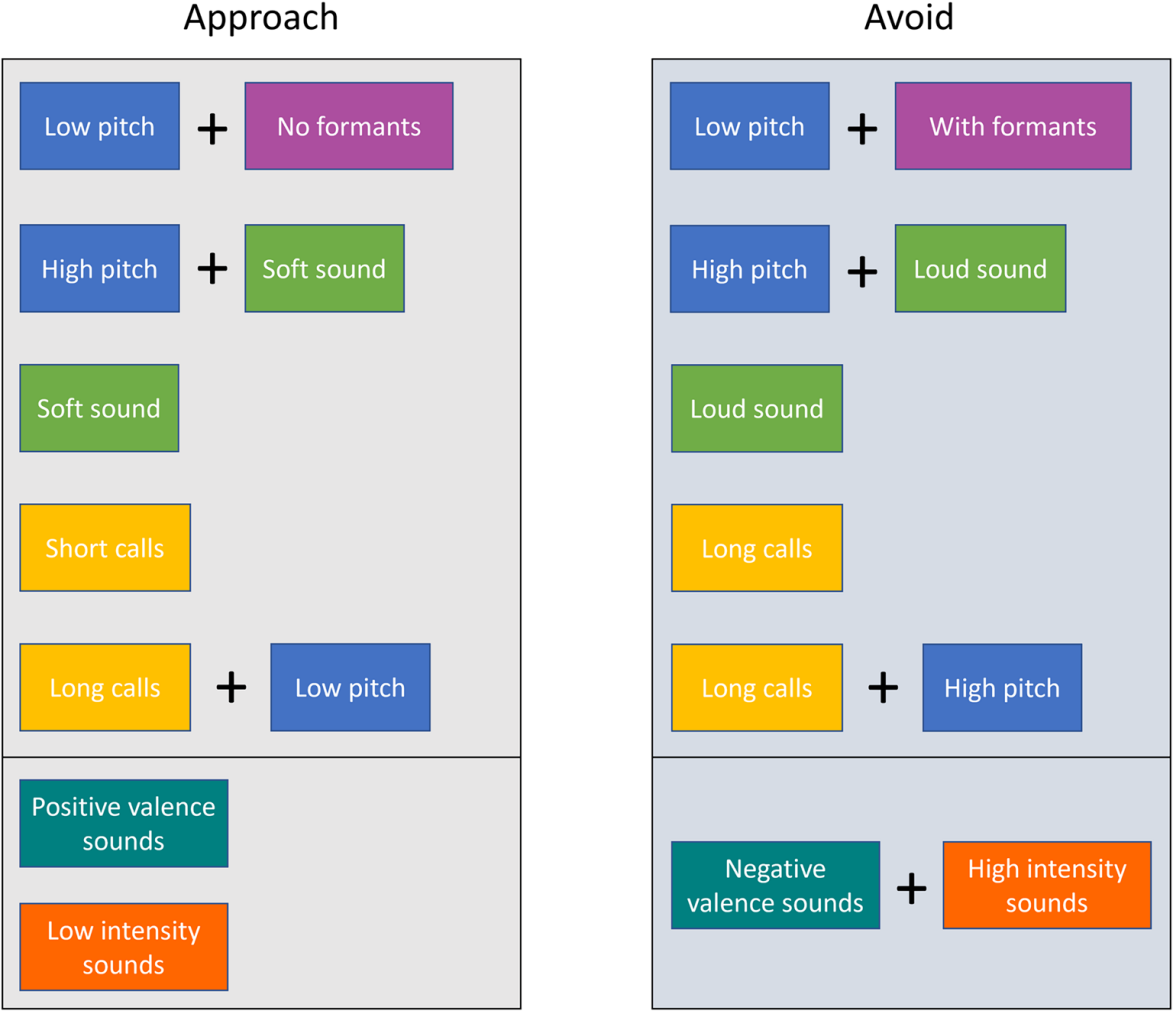
A summary of effects of the acoustic features and emotional scales on approach and avoidance. The effects of acoustic features are indicated in the upper sections of the approach and avoidance columns by different colour backgrounds, pitch (blue); loudness (green), call length (yellow), formants (purple). In the bottom section of the columns the emotional scale effects are shown, valence (teal), intensity (orange). Interactions between effects are indicated with positive (+) sings.

Regarding our first question, the results show that the elicited approach-avoidance reactions are linked with acoustic features such as fundamental frequency (f_0_), call length and loudness, which parameters frequently affect approach-avoidance reactions in interaction with each other, as well as with the presence of acoustic features characteristic of animal vocalisations (Fig. 9). Following our prediction, sounds composed of short calls were associated with approach, and louder sounds elicited avoidance in most complexity categories, even though the effect of softer sounds was more prevalent in eliciting approach. In contrast, pitch affected the approach-avoidance reactions in a more complex manner than predicted, acting in interaction with other features.

The acoustic features of pitch and loudness enable the distinction of loud and high-pitched calls from the set of generated artificial sounds, which were rated as more aversive, thus—besides their acoustical parallels—also making them functionally similar to alarm calls^59,64^ or scream-like vocalizations^65^. In contrast, the high-pitched but less loud artificial sounds are akin to human and non-human cries^66^, again providing a biologically rooted functional analogue: the softer and high-pitched artificial sounds had an approach-eliciting effect similarly to cry vocalizations.

Concerning our second question, we have found that the approach-avoidance reactions were linked to the perceived valence and intensity of the sounds. The associations of the valence and intensity scores with the social dimension corroborate the distinction of sounds reminiscent of cry and alarm vocalisations, as alarm calls are usually emitted in high arousal negative valence inner states, while the intensity of the emitter’s inner state during cry-like vocalisations tend to be less intense. We see the same pattern in case of the artificial sounds, as sounds rated to have high arousal and negative valence functioned as alarm calls, eliciting avoidance, and less intense sounds had corresponding approach-eliciting reactions as cries.

In line with earlier findings^29,37,67^ suggesting an association of shorter call lengths with positive inner states and playful or comfortable social contexts, here we also found that shorter calls are more likely to evoke approach reaction. Although our knowledge about animal vocalizations with positive valence is limited^28^, it is accepted that these calls have a social function of eliciting and maintaining the given positive interaction, thus ‘approach’ and ‘stay’ are certainly adaptive reactions. Accordingly, the artificial sounds which were associated with positive valence earlier, indeed more readily evoked approach from the participants. Applying calls with such characteristics might have an importance in social robotics in making artificial agents more acceptable and appealing to humans.

Interestingly, we have not found any demographic effects. Previously, age had been found to affect the valence and intensity ratings of artificial sounds, as older participants rated these sounds overarchingly as more positive and less intense than younger participants^31^. This suggests that such age-related positivity effect^68^ is independent from the perception of the social information encoded in the acoustic structure of vocalizations. As we did not find a connection with age or gender on the effects of the social dimension, we can assume that the approach-avoidance reactions are similarly elicited in various demographic groups, providing universally usable functional sounds for robots, and it is also likely that the cultural background has also a small effect on the variability of the approach-avoidance reaction.

As the approach-avoidance reactions of participants differed when listening to the artificial sounds according to various acoustic features, we can propose a set of specific acoustic features for sounds with different communicative functions for social robots, summarised in Fig. 9. As a main point, we recommend the use of short, soft sounds for the function of eliciting approach, and long, loud, high pitched sounds for scenarios in which withdrawal from the robot is needed. We only recommend low pitched sounds for avoidance if the sounds have a harsh (wide frequency band) quality, otherwise low pitch is more likely to evoke approach.

The emotionally expressive artificial sounds presented in the current article could contribute to the contagion of emotions via vocal communication, as seen in animals^8^ and humans^69^. This might be an important factor to enhance the acceptability of robots as social interaction partners and promote their embedment in the human environment. As emotion contagion enables group coordination and the synchronisation of inner states^8^, we consider it a valuable possible tool for HRI and collaborative robotics.

A limitation of our study is the relative lack of sounds corresponding to frequency band features of agnostic vocalisations: as we have utilised the same sound samples of our previous study^31^, we have not modulated the noisiness of the generated sounds. Noisy, harsh sounds with wide frequency bands with long call lengths and low frequencies are expected to be perceived as repellent, eliciting avoidance, according to the motivation-structural rules^43^. As we have seen, in the complex sound categories—in which formant modelling was included as well—low frequency sounds elicited avoidance, in contrast with other sound categories. The modelling of formants might have affected the perception of these sounds, causing them to be perceived noisier, and hence reversing the effect of pitch.

In addition to the explored simple rules based on animal vocalisations, other complex influencing factors could be considered in HRI, such as cultural factors or expectations towards robots, which could be taken into account in extending the non-verbal acoustic communication of artificial agents with sound signals that can be interpreted by humans as e.g., questioning-affirming or approving-disapproving.

In conclusion, the complexity of our results indicates the need for multidimensional emotional models to be implemented in social robots^70^. These emotional models should be implemented with a set of basic rules, regulating the main connections between inner states and emotion expressions. Inputs should be provided by perceived and processed environmental and inner (operational, e.g., battery charge level) stimuli. Thus, instead of using single or combined pre-recorded vocalisations as output, social robots should be able to produce situation-specific unique blended sounds generated in situ by the emotional model. The rules of the model could be fine-tuned by the reactions of the human partner via machine learning methods, providing refinement of the emotion expressions. Such learning capacity could enable the continuous adaptation of the robot to its specific social and cultural environment. Our future work will focus on spontaneous human–robot interactions to further investigate and utilise the created artificial sounds in more ecologically valid situations, via autonomous robots operating in e.g., restaurants or office environments.

### Supplementary Information

Supplementary Information 1.

Supplementary Information 2.

Supplementary Information 3.

## Data Availability

The dataset generated during the current study is available as a supplementary file (Dataset.csv).
