# Supplementary material for: People follow motivation-structural rules when they react to synthetised sounds
Source: Sci Rep. 2024 Jul 26;14:17656. doi: 10.1038/s41598-024-68165-5 (PMC11291635; doi:10.1038/s41598-024-68165-5)
Supplement: Supplementary file 3 — Supplementary Information 3. [file 41598_2024_68165_MOESM3_ESM.pdf]

Supplementary results

People follow motivation-structural rules when they react to synthetised sounds

Beáta Korcsok[a*,**], Tamás Faragó[b,**], Bence Ferdinandy[a], Ádám Miklósi[a,c], Péter Korondi[d], Márta Gácsi[a,c]

[a]*HUN-REN–ELTE Comparative Ethology Research Group, Budapest, Hungary*
[b]*Neuroethology of Communication Lab, Department of Ethology, Eötvös Loránd University, Budapest, Hungary*
[c]*Department of Ethology, Eötvös Loránd University; Budapest, Hungary*
[d]*Department of Mechatronics, Faculty of Engineering, University of Debrecen, Debrecen, Hungary*

**Corresponding author: korcsokbea@gmail.com*
***Equal contribution*

S1

	Model1: Demographic effects			
Predictors	*Odds Ratios*	*CI*	*Statistic*	*p*
far|med far	0.180	0.128 – 0.252	-9.921	**<0.001**
med far|stay	0.531	0.379 – 0.743	-3.684	**<0.001**
stay|med close	1.158	0.827 – 1.621	0.854	0.393
med close|close	4.466	3.184 – 6.264	8.670	**<0.001**
age s	1.160	0.951 – 1.414	1.467	0.142
language [hu]	1.345	0.903 – 2.003	1.456	0.145
sex [male]	1.030	0.686 – 1.544	0.141	0.888
side [1]	0.949	0.865 – 1.041	-1.116	0.264
**Random Effects**				
σ^2	3.29			
$\tau_{00\ soundid}$	1.06			
$\tau_{00\ subj}$	1.53			
ICC	0.44			
N_{subj}	172			
$N_{soundid}$	343			
Observations	6824			
Marginal R^2 / Conditional R^2	0.007 / 0.445			

Supplementary Table S1
Mixed effects ordinal regression model, testing demographic (age, gender) and set-up related (side of the stimulus, survey language) effects. The participant ID and sound ID are included as random intercepts.

S2

	Model2: Acoustic features			
Predictors	*Odds Ratios*	*CI*	*Statistic*	*p*
far|med far	0.092	0.068 – 0.125	-15.236	**<0.001**
med far|stay	0.273	0.202 – 0.370	-8.391	**<0.001**
stay|med close	0.597	0.441 – 0.807	-3.348	**0.001**
med close|close	2.288	1.690 – 3.097	5.357	**<0.001**
f0 s	0.668	0.585 – 0.763	-5.942	**<0.001**
cl s	0.790	0.633 – 0.986	-2.082	**0.037**
type2 × Pulse train	0.124	0.063 – 0.244	-6.031	**<0.001**
type3 × Pitch down	1.631	1.217 – 2.186	3.277	**0.001**
type4 × Pitch up	0.923	0.693 – 1.227	-0.553	0.580
type5 × Variable	0.987	0.744 – 1.308	-0.093	0.926
type6 × Complex down	0.532	0.410 – 0.690	-4.749	**<0.001**
type7 × Complex up	0.324	0.249 – 0.421	-8.425	**<0.001**
loud s	0.598	0.394 – 0.908	-2.412	**0.016**
f0 s × type2 × Pulse train	1.079	0.766 – 1.520	0.435	0.663
f0 s × type3 × Pitch down	1.203	0.997 – 1.452	1.932	0.053
f0 s × type4 × Pitch up	1.011	0.835 – 1.225	0.116	0.908
f0 s × type5 × Variable	1.082	0.888 – 1.319	0.781	0.435
f0 s × type6 × Complex down	1.931	1.548 – 2.409	5.836	**<0.001**
f0 s × type7 × Complex up	2.141	1.703 – 2.691	6.528	**<0.001**
cl s × type2 × Pulse train	1.087	0.701 – 1.685	0.374	0.709
cl s × type3 × Pitch down	0.866	0.661 – 1.135	-1.042	0.297
cl s × type4 × Pitch up	0.847	0.650 – 1.105	-1.224	0.221
cl s × type5 × Variable	0.889	0.676 – 1.167	-0.848	0.396
cl s × type6 × Complex down	0.837	0.626 – 1.120	-1.196	0.232
cl s × type7 × Complex up	0.718	0.541 – 0.951	-2.306	**0.021**
f0 s × cl s	0.869	0.806 – 0.936	-3.689	**<0.001**
f0 s × loud s	0.753	0.679 – 0.834	-5.450	**<0.001**
cl s × loud s	1.127	0.971 – 1.308	1.573	0.116
(type2 × Pulse train) × loud s	0.882	0.524 – 1.486	-0.470	0.638

(type3 × Pitch down) × loud s	0.601	0.342 – 1.054	-1.776	0.076
(type4 × Pitch up) × loud s	0.488	0.284 – 0.838	-2.599	**0.009**
(type5 × Variable) × loud s	1.066	0.614 – 1.852	0.228	0.820
(type6 × Complex down) × loud s	0.671	0.398 – 1.132	-1.496	0.135
(type7 × Complex up) × loud s	0.582	0.350 – 0.968	-2.085	**0.037**
**Random Effects**				
σ^2	3.29			
$\tau_{00\ soundid}$	0.05			
$\tau_{00\ subj}$	1.58			
ICC	0.33			
N_{subj}	172			
$N_{soundid}$	343			
Observations	6824			
Marginal R^2 / Conditional R^2	0.170 / 0.446			

Supplementary Table S2
Mixed effects ordinal regression model, testing acoustic effects (call length, pitch, loudness, sound category and their two-way interactions). The participant ID and sound ID are included as random intercepts.

S3

contrast	*estimate*	*conf.low*	*conf.high*	*statistic*	*adj.p.value*
1: Sine wave - 2: Pulse train	-0.076	-0.591	0.439	-0.435	0.999
1: Sine wave - 3: Pitch_down	-0.185	-0.468	0.097	-1.932	0.459
1: Sine wave - 4: Pitch_up	-0.011	-0.3	0.277	-0.116	1
1: Sine wave - 5: Variable	-0.079	-0.377	0.219	-0.781	0.987
1: Sine wave - 6: Complex_down	-0.658	-0.991	-0.326	-5.836	**0**
1: Sine wave - 7: Complex_up	-0.761	-1.105	-0.417	-6.528	**0**
2: Pulse train - 3: Pitch_down	-0.109	-0.632	0.414	-0.615	0.996
2: Pulse train - 4: Pitch_up	0.065	-0.46	0.59	0.364	1
2: Pulse train - 5: Variable	-0.003	-0.533	0.527	-0.016	1
2: Pulse train - 6: Complex_down	-0.582	-1.119	-0.045	-3.195	**0.024**
2: Pulse train - 7: Complex_up	-0.685	-1.229	-0.142	-3.716	**0.004**
3: Pitch_down - 4: Pitch_up	0.174	-0.125	0.472	1.717	0.604
3: Pitch_down - 5: Variable	0.106	-0.202	0.414	1.016	0.951
3: Pitch_down - 6: Complex_down	-0.473	-0.814	-0.132	-4.088	**0.001**
3: Pitch_down - 7: Complex_up	-0.576	-0.928	-0.224	-4.823	**0**
4: Pitch_up - 5: Variable	-0.068	-0.381	0.246	-0.637	0.996
4: Pitch_up - 6: Complex_down	-0.647	-0.994	-0.3	-5.502	**0**
4: Pitch_up - 7: Complex_up	-0.75	-1.107	-0.393	-6.189	**0**
5: Variable - 6: Complex_down	-0.579	-0.933	-0.226	-4.829	**0**
5: Variable - 7: Complex_up	-0.682	-1.046	-0.319	-5.536	**0**
6: Complex_down - 7: Complex_up	-0.103	-0.49	0.284	-0.784	0.987

Supplementary Table S3
Post-hoc pairwise comparisons of sound category and pitch interactions.

S4

contrast	*estimate*	*conf.low*	*conf.high*	*statistic*	*adj.p.value*
1: Sine wave - 2: Pulse train	0.125	-0.659	0.909	0.47	0.999
1: Sine wave - 3: Pitch_down	0.509	-0.336	1.355	1.776	0.564
1: Sine wave - 4: Pitch_up	0.718	-0.097	1.533	2.599	0.126
1: Sine wave - 5: Variable	-0.064	-0.895	0.766	-0.228	1
1: Sine wave - 6: Complex_down	0.398	-0.387	1.184	1.496	0.748
1: Sine wave - 7: Complex_up	0.542	-0.224	1.308	2.085	0.361
2: Pulse train - 3: Pitch_down	0.384	-0.396	1.165	1.451	0.774
2: Pulse train - 4: Pitch_up	0.593	-0.154	1.34	2.34	0.225
2: Pulse train - 5: Variable	-0.189	-0.951	0.572	-0.733	0.991
2: Pulse train - 6: Complex_down	0.273	-0.431	0.977	1.144	0.914
2: Pulse train - 7: Complex_up	0.417	-0.255	1.088	1.829	0.528
3: Pitch_down - 4: Pitch_up	0.209	-0.605	1.022	0.755	0.989
3: Pitch_down - 5: Variable	-0.574	-1.405	0.258	-2.035	0.393
3: Pitch_down - 6: Complex_down	-0.111	-0.9	0.678	-0.415	1
3: Pitch_down - 7: Complex_up	0.032	-0.735	0.799	0.124	1
4: Pitch_up - 5: Variable	-0.782	-1.582	0.017	-2.885	0.06
4: Pitch_up - 6: Complex_down	-0.32	-1.074	0.435	-1.249	0.875
4: Pitch_up - 7: Complex_up	-0.176	-0.907	0.554	-0.712	0.992
5: Variable - 6: Complex_down	0.463	-0.305	1.23	1.777	0.564
5: Variable - 7: Complex_up	0.606	-0.143	1.354	2.387	0.204
6: Complex_down - 7: Complex_up	0.143	-0.553	0.84	0.607	0.997

Supplementary Table S4
Post-hoc pairwise comparisons of sound category and loudness interactions.

S5

	Model3: Emotional scale associations			
Predictors	*Odds Ratios*	*CI*	*Statistic*	*p*
far|med far	0.144	0.116 – 0.180	-17.246	**<0.001**
med far|stay	0.429	0.345 – 0.532	-7.688	**<0.001**
stay|med close	0.936	0.755 – 1.161	-0.599	0.549
med close|close	3.590	2.890 – 4.460	11.544	**<0.001**
int s	0.869	0.790 – 0.956	-2.879	**0.004**
val s	2.098	1.901 – 2.315	14.740	**<0.001**
int s × val s	1.164	1.071 – 1.266	3.560	**<0.001**
**Random Effects**				
σ^2	3.29			
$\tau_{00\ soundid}$	0.32			
$\tau_{00\ subj}$	1.58			
ICC	0.37			
N_{subj}	172			
$N_{soundid}$	343			
Observations	6824			
Marginal R^2 / Conditional R^2	0.126 / 0.446			

Supplementary Table S5
Mixed effects ordinal regression model, testing valence and intensity effects. The participant ID and sound ID are included as random intercepts.
